# Cross-Frequency Power-Power Coupling Analysis: A Useful Cross-Frequency Measure to Classify ICA-Decomposed EEG

**DOI:** 10.3390/s20247040

**Published:** 2020-12-09

**Authors:** Nattapong Thammasan, Makoto Miyakoshi

**Affiliations:** 1 Human Media Interaction, Faculty of Electrical Engineering, Mathematics and Computer Science, University of Twente, 7522 NB Enschede, The Netherlands; n.thammasan@utwente.nl; 2 Swartz Center for Computational Neuroscience, Institute for Neural Computation, University of California San Diego, La Jolla, CA 92093, USA

**Keywords:** EEG, MEG, fourier transform, cross-frequency coupling, comodulogram, comodugram, independent component analysis

## Abstract

Magneto-/Electro-encephalography (M/EEG) commonly uses (fast) Fourier transformation to compute power spectral density (PSD). However, the resulting PSD plot lacks temporal information, making interpretation sometimes equivocal. For example, consider two different PSDs: a central parietal EEG PSD with twin peaks at 10 Hz and 20 Hz and a central parietal PSD with twin peaks at 10 Hz and 50 Hz. We can assume the first PSD shows a mu rhythm and the second harmonic; however, the latter PSD likely shows an alpha peak and an independent line noise. Without prior knowledge, however, the PSD alone cannot distinguish between the two cases. To address this limitation of PSD, we propose using *cross-frequency power–power coupling* (PPC) as a post-processing of independent component (IC) analysis (ICA) to distinguish brain components from muscle and environmental artifact sources. We conclude that post-ICA PPC analysis could serve as a new data-driven EEG classifier in M/EEG studies. For the reader’s convenience, we offer a brief literature overview on the disparate use of PPC. The proposed cross-frequency *power–power coupling analysis toolbox* (PowPowCAT) is a free, open-source toolbox, which works as an EEGLAB extension.

## 1. Introduction

### 1.1. Background

Power spectral density (PSD) calculations using fast Fourier transform (FFT) are ubiquitous in magnetoencephalography (MEG) and electroencephalography (EEG) analyses. However, this approach’s drawback is the lack of temporal resolution; hence, sliding window solutions such as short-term Fourier Transform and wavelet transform become necessary for temporal resolution.

*Cross-frequency power–power coupling* (PPC) addresses the temporal resolution dilemma by calculating spectral covariance. When the amplitudes of two oscillations in different frequencies covaries along with time, PPC occurs. The resulting spectral covariance is a square matrix plot (also called a *comodulogram* [1,2,3,4,5,6,7,8] or a *comodugram* [9,10,11,12,13,14,15]. The following examples illustrate two different types of cross-frequency coupling, the distinction of which is of common interest in EEG and MEG studies. Imagine two peaks occurring in a PSD plot at X Hz and at Y Hz. Power changes in the two oscillations causing the two peaks may or may not be correlated. Consider the first case where a PSD calculated from data recorded on or adjacent to the sensorimotor area shows a mu rhythm with the first peak within the alpha band and the second harmonic in the beta band (similar to the Greek alphabet letter “µ” that has two peaks). However, these twin peaks do not reflect independent processes because the two oscillations are phase-locked [16]. Consider the second case that a PSD has twin peaks at the alpha band and at a typical power-line frequency band (50 or 60 Hz). These two PSD peaks reflect separate processes with no temporal correlation between the sources. Distinguishing these two cases is essential in interpreting M/EEG PSD data. Evaluating the PSD alone without incorporating temporal information potentially makes it harder for EEG researchers to correctly identify and evaluate different processes embedded within the PSD. Use of the PPC to analyze two peak frequencies within a single PSD helps to interpret the data accurately. However, as far as we know, the PPC has been under-utilized in the field of M/EEG research. Past PPC publications are sporadic, found in different fields of electrophysiology, cognitive, and clinical neurophysiology minimal references to each other. Overall, given PPC’s ability to incorporate temporal information missing from the PSD has been grossly underutilized and poorly recognized. To address this situation, and before we discuss this study’s goals, we briefly summarize the literature of PPC from subfields of electrophysiology.

### 1.2. Mini-Review on History of Power–Power Coupling Analysis

The frequency-by-frequency square matrix plot that represents cross-frequency PPC has been called *comodulogram* [1,2,3,4,5,6,7,8], sometimes *comodugram* [9,10,11,12,13,14,15]. The cross-frequency PPC was initially applied to MEG data from human subjects to define chronic neurological disorders such as Parkinson’s disease [17,18] and neuropsychiatric diseases including neurogenic pain, depression, Parkinson’s disease, and schizophrenia, caused by brain thalamocortical dysrhythmia [19]. The cross-frequency PPC was also measured in LFP data acquired from hippocampus, postsubiculum, and dorsocentral striatum of behaving rats to identify biologically generated fundamental frequencies [20] and from hippocampus of locomotor behaving rats to investigate synchrony of delta and theta power [21]. The comodulogram was also used in qEEG analyses to topographically quantify the temporal synchrony of EEG power fluctuations between recording sites in order to discover regions where functional homogeneity or heterogeneity deviate from normal. Clinically significant patterns of the altered functional interactions were found in epileptic patients after left anterior temporal lobectomies and patients with nearly complete surgical transection of the corpus callosum [1]. Recently, comodulogram was used to analyze beta–gamma coupling of field potential recorded in rat motor thalamus during a forced-choice task [2], broad-band theta couplings of LFPs in rat visual cortex during waking, REM (rapid eye movement), and non-REM [3], coupling in ripples (140–220 Hz) oscillations of LFP in rat posterior parietal cortex and midline structure during non-REM sleep following learning [4], and anti-coupling between beta and high gamma (80 Hz) oscillations of LFPs in rat basal ganglia during controlled movement [5]. Recent articles reported that comodulogram distinguished leader rats from follower rats by showing different levels of delta–theta coupling of LFPs in right pre-limbic cortices during platform climbing task [6], identifying three independently varying frequency bands of LFPs in rat frontal cortical areas during REM, non-REM, and micro-arousal sleep [7], and identified fundamental frequencies of LFPs in rat medial entorhinal cortex during running [8]. During the early 2000’s, the same technique, *now called slightly differently, comodugram*, was used to analyze broad-band frequencies of LFPs recorded in mouse hippocampus [9,10], theta–gamma coupling of LFPs in rat entorhinal cortex [11], coupling between ripples (140–220 Hz) and fast gamma (90–140 Hz) oscillations of LFPs in rat hippocampus recorded during movement [12], coupling between slow and fast gamma oscillations of LFPs in mouse basolateral amygdala during fear expression [13], and gamma coupling of LFPs between hippocampus and prefrontal cortex of rats in anesthesia and natural sleep [14]. As for behavioral relevance, the coupling between theta (4–10 Hz) and low gamma (30–50 Hz) powers of LFPs in rat hippocampus predicted successful memory retrieval [15]. The analysis of matrix of cross-frequency PPC was also used in revealing theta–gamma (50–130 Hz) coupling [22] and fundamental frequencies [23] of LFPs in rat hippocampus. Recent studies on humans reporting cross-regional PPC using EEG [24] and MEG [25]. Despite the increasing interest in animal studies, particularly in human EEG studies, the cross-frequency PPC analysis remains underutilized given the potential usefulness and versatility.

### 1.3. Goal of the Current Study

The study’s goal is to demonstrate the usefulness of cross-frequency PPC measure within EEG data analysis, specifically as a post-independent component analysis (ICA) [26,27,28,29,30]. One of the post-ICA processing challenges is classifying the obtained independent components (ICs). Many applications aim for automated labeling of the ICs including MARA [31,32], ADJUST [33], FASTER [34], eyeCatch [35], IC MARC [36], SASICA [37], ICLabel [38], and others [39,40]. Multiple solutions exist because IC classification is a difficult task. The properties of ICA-decomposed EEG data may be represented by event-related potential (ERPs), event-related power-spectral perturbation, inter-trial phase coherence (ERSP and ITC [41]), scalp topography, and dipole locations. Adding different types of EEG property that carry unique information not represented by these cited measures improves the characterization of the obtained ICA-decomposed EEG data. Here, we demonstrate that brain, eye, and muscle classes of ICA-decomposed scalp-recorded signals have characteristic PPC readily visible in comodulograms. The visible features of PPC provide complimentary temporal information useful for classification and labeling of ICs. The proposed method is available as a plugin *Power–Power Coupling Analysis Tool* (*PowPowCAT*) (https://sccn.ucsd.edu/wiki/Power-Power_Coupling_Analysis_Tool). The plugin is for a free, open-source library EEGLAB [42].

## 2. Materials and Methods

### 2.1. Data Acquisition and Preprocessing

The EEG data used is from a previously published study on facial familiarity [43]. Twenty-four paid volunteers participated in the study (mean 21.1 ± 1.4 years old, range 20–26, 11 women). All participants gave written informed consent. All experimental procedures followed the Declaration of Helsinki. Visual stimuli with three levels of familiarity (self-relevant, familiar, and unfamiliar) and with two levels of categories (face and cup) were presented. The participant’s task was to judge the level of object familiarity by pressing one of three buttons as quickly as possible. The stimulus was presented either unilaterally (to the left or right visual hemifield) or bilaterally (identical images presented to both visual hemifields). During the task, EEG signals were recorded using the Nihon Koden EEG 1100 (Tokyo, Japan) with 33 electrodes (AFp9, Fp1, Fp2, Fz, F3, F4, F7, F8, FC1, FC2, FC5, FC6, Cz, C3, C4, T7, T8, CP1, CP2, CP5, CP6, TP9, TP10, Pz, P3, P4, P7, P8, O1, O2, PO9, PO10, and Iz), positioned in correspondence with international 10–10 system, at the sampling frequency of 500 Hz, which was later downsampled to 200 Hz. Electrode impedance was kept typically below 5 kΩ. EEG electrodes were initially referenced to a nose tip, and later, a common average re-referencing was performed to gain better estimates of reference-independent potentials [44]. An FIR high-pass filter of 0.5 Hz @ −6dB (transient bandwidth 1 Hz, Hamming windowed) [45] was applied to the continuous EEG data. Finally, adaptive mixture ICA [46] was performed to obtain 32 ICs from each of the 24 datasets as it is most efficient among all decomposition methods in reducing mutual information in the component time courses [47]. For each scalp topography of ICs, which are the column of the mixing matrix (i.e., the inverse of the weight matrix), the estimated equivalent current dipole was fit using Dipfit2.2. Dipfit2.2 is a group of functions taken from Fieldtrip Toolbox [48]. It uses a four-shell spherical head model co-registered to each subject’s electrode locations by warping the electrode locations to the template head from Montreal Neurological Institute (MNI).

We chose ICA as a preprocessing technique. ICA relies on these three assumptions: (1) *Functional independence* of cortical regions should be represented by *temporal independence* of the local field potentials; (2) volume conduction (i.e., signal paths from an EEG source to scalp sensors) and signal mixing process are *linear* and *instantaneous*; and (3) the functional mapping of the neocortex is *stationary* across the recording. If these assumptions hold, the ICA’s spatial filter produces (1) effective sources of EEG as temporally maximally independent signals as well as (2) their linear and instantaneous mixing processes (i.e., volume conduction) that are (3) stationary. The beauty of ICA in application to scalp-recorded EEG is that ICA solves the spatial problem without requiring any spatial information. ICA does not require either electric forward model of the human head or knowledge of scalp channel locations. Onton and Makeig [30] established the physiological interpretation of ICA on EEG. The only confirmed case of violation of the nonstationary assumption is traveling wave, which is thought of as moving EEG sources in the brain. In [30], the authors simulated that a traveling wave spreading across 3 cm of distance with the speed of 2 m/s oscillating in 10 Hz produces 27 degrees of phase shift, which is only 7.5% of a single cycle of 10 Hz. They determined that this level of phase shift could be trivial and regarded as nearly synchronous for ICA. However, traveling waves with larger scales, such as the one reported by Muller and colleagues [49], would result in failure in applying ICA. Nonetheless, previous studies on EEG experiments during awake states reported the successful application of ICA [28,29,30,50,51,52].

For the group-level data integration, independent component (IC) clustering using the k-means algorithm, implemented in EEGLAB STUDY for grouping similar ICs across subjects, after PCA-dimension reduction was used. The equivalent current dipoles were normalized with moments and the weight factor was empirically determined as 5. PSD and scalp topography were also precomputed for each IC, and they were dimension-reduced to 10 for each using PCA. Next, k-means clustering was applied on the dimension-reduced data feature vectors to categorize 790 ICs into 31 clusters (the number of clusters was based on [53]). Each cluster was examined to label as brain, muscle, eye, and other noise by visual inspection on PSD, equivalent current dipole locations, and scalp topography. As a result, 5 of 31 clusters were identified as eye-movement IC clusters for their locations near the edge of ventral frontal brain regions and for their PSD being relatively high at low frequencies with no spectral peak; 7 of 31 clusters were identified as clusters associated with muscle artifacts for their locations outside of the brain or near lower head region and for their characteristic mean spectral plateau above 25 Hz. Thus, 16/31 clusters were identified as clusters associated with brain activity. They showed PSD feature of the 1/f characteristic typical in EEG signals sometimes with peaks in the alpha band (8–13 Hz). Finally, 3/31 clusters were excluded from the analysis as outliers after manually cleaning the other clusters by gathering cluster-inconsistent ICs due to limitations of the algorithm in clustering accuracy. Based on this initial classification, we combined each class of clusters to form aggregated clusters of brain activities (consisting of 380 ICs: mean 15.83 ± 3.60 ICs from each subject), eye movements (consisting of 87 ICs: mean 3.63 ± 1.47 ICs from each subject), and muscle activities (consisting of 213 ICs: mean 8.88 ± 3.66 ICs from each subject), and outliers (consisting of 110 ICs: mean 4.58 ± 1.82 ICs from each subject). The cross-frequency PPC of each IC was computed and studied separately in each class.

### 2.2. Assessment of Cross-Frequency Power–Power Coupling

On continuous IC time-series data, Short-Term Fourier Transform (STFT) with a 50%-overlapping 1-s sliding window was applied to compute spectrograms using Matlab’s *spectrogram* function to generate semi-logarithmically spaced 1–50 Hz divided into 100 frequency bins. Additionally, 20% of the individual PSD windows were excluded using distance from median values. This manner of cleaning the data preprocessing is justified in the Discussion section.

The correlation between the power spectrum S(f) at a frequency $f_{i}$ and at another frequency $f_{j}$ can be calculated using the expression

(1) $$corr_{ij}\;=\;\frac{\sum_{k}\left(S_{k}\left(f_{i}\right)-\bar{S\left(f_{i}\right)}\right)\left(S_{k}\left(f_{j}\right)-\bar{S\left(f_{j}\right)}\right)}{\sigma_{i}\sigma_{j}},$$

where $S_{k}\left(f_{i}\right)$ is the PSD at frequency $f_{i}$ in time-window *k*, $\bar{S\left(f_{i}\right)}$ the averaged PSD at frequency $f_{i}$ overall sliding windows, $\sigma_{i}$ the standard deviation of the PSD at frequency $f_{i}$, and *k* ranges over all sliding windows.

To illustrate common patterns in comodulograms in each class, we generated a standardized matrix from correlation matrices of all ICs in the class. Given that a class contains m ICs, the standardized correlation at the frequency $f_{i}$ and at another frequency $f_{j}$, namely $z_{ij}$, is computed as

(2) $$z_{ij}\;=\;\frac{\bar{corr_{ij}}}{\sigma_{ij}},$$

where $\bar{corr_{ij}}\;=\;\frac{\sum_{m}corr_{ij}}{m}$ is the averaged correlation across all m ICs at the frequency $f_{i}$ and at another frequency $f_{j}$, and $\sigma_{ij}$ is the standard deviation of correlation. The coupling of any frequencies that is commonly found across ICs in the class is expected to be salient in the standardized matrix.

### 2.3. Visualizations by Power–Power Coupling Analysis Toolbox (PowPowCAT)

**Figure 1:**
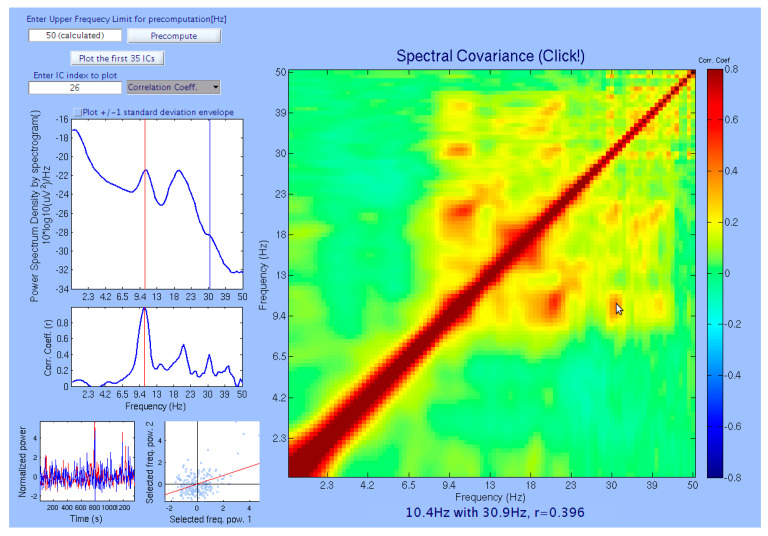
The main window of the toolbox that let users choose the cross-frequency points interactively by moving the mouse cursor over the right panel: currently, the cursor is pointing to the crossing point of 10.4 Hz and 30.9 Hz.

The matrix of correlation coefficients computed from a representative recording is shown in Figure 1. This figure is a screen capture from our in-house developed *PowPowCAT* (ver 1.00) graphical user interface (GUI). In the right large plot, the color scale shows that red indicates positive coupling (correlation), and the blue indicates negative coupling (anti-correlation). This panel also serves as an interactive interface to select two frequencies by moving the mouse cursor over the frequencies’ desired combination. The top left panel shows the mean PSD across all 1-s sliding windows, as well as its standard deviations. The middle left panel shows the vertical profile of the color map on the right large plot. The bottom left small panel shows the downsampled and normalized time-series of the PSD of the selected pair of the frequencies. The bottom right small panel shows the scatter plot of the downsampled data points shown in the bottom left small panel.

To test the null hypothesis that the power spectra time series of two different frequencies, $f_{i}$ and $f_{j}$, are not coupled in the data, non-parametric surrogate data method is supported as it makes minimum assumptions. It preserves the original data’s statistical properties while generating time series that are randomized such that any possible nonlinear coupling is removed [54]. It has also been actively used to test coupling in nonlinear systems in many EEG studies [55,56]. Specifically, the surrogate data method randomizes time-window *k* differently for each frequency bin to build surrogate time-frequency time series and computes surrogate cross-frequency PPC. This process is repeated 5000 times (default) to produce distributions for the dataset in which the null hypothesis holds, i.e., the chance-level of the cross-frequency PPC. The original, non-permuted data are then compared to the surrogate distribution to obtain uncorrected *p*-values. The significance threshold can be selected to be either 0.05 or 0.01 after false discovery rate (FDR) correction across all ICs and all pairs of frequencies. Pixels with non-significant results are masked.

### 2.4. Clustering ICs Using Comodulogram as Clustering Criterion

To test whether comodulogram is useful for clustering the data, we re-clustered the ICs using their comodulograms as the clustering criterion (k-means algorithm without PCA-dimension reduction) to see if the clusters show physiologically meaningful source distributions. The three major classes of the scalp recorded signals, brain, eye, and muscle classes, have characteristic source distributions. Brain sources should be localized within the brain; eye sources within the peri-ocular regions; and muscle sources in the periphery of the anterior, temporal, and occipital regions.

After computing comodulograms of all 790 ICs, the non-redundant parts of the comodulograms were used as feature vectors. PCA dimension reduction was not applied for this analysis. In performing the same k-means algorithm, we set the number of clusters to be 8 to give it sufficient degrees of freedom to represent at least three classes of physiological signals (brain, eye, and muscle) plus noise. For each resulting cluster, the standardized correlation matrix was calculated from cluster members’ ICs using Equation (2). In addition, the corresponding brain regions were illustrated by calculating the probabilistic density of the equivalent current dipoles using *std_dipoleDensity* plugin for EEGLAB that implemented 3-D Gaussian kernel transformation with full-width half maximum (FWHM) of 20 mm [47].

To test further if the comodulogram-based clustering produced reasonable classifications of data, we used *ICLabel* [38] to classify ICs into classes and then evaluated the proportion of ICs classes in each cluster resulted from comodulogram-based clustering. The ICLabel is an open-source, freely available plugin for EEGLAB that serves as a state-of-the-art IC classification tool. The classification model was trained on a large-scale ICs collection using a set of sophisticated features and labels given by human experts. It provides probabilistic class labels of Brain, Muscle, Eye, Heart, Line Noise, Channel Noise, and Others, and the highest class-label probability determines the class of that IC. We adopted this tool to generate the class labels for ICs. Thus, ICLabel served as the operational *ground truth* generator in the current study. We examined the percentage of the most prevailing class labels per cluster where higher probability indicates higher consistency with the operational ground truth. It should also be consistent with our visual evaluation on standardized comodulogram and probabilistic dipole density. The percentage of the most prevailing class label per cluster is called *consistency level,* indicating consistency between the results from ICLabel and our proposed comodulogram-based clustering. Moreover, among the total 790 ICs, we calculated the percentage of ICs classified by ICLabel into each class, regardless of the cluster, to deliver the distribution of classes. We found that the most prevalent class label is the brain class, and the percentage of this class was 42.5%. We adopted this number as the chance level of classification for comparison.

## 3. Results

### 3.1. Standardized Comodulograms for Brain, Eye, and Muscle Classes

**Figure 2:**
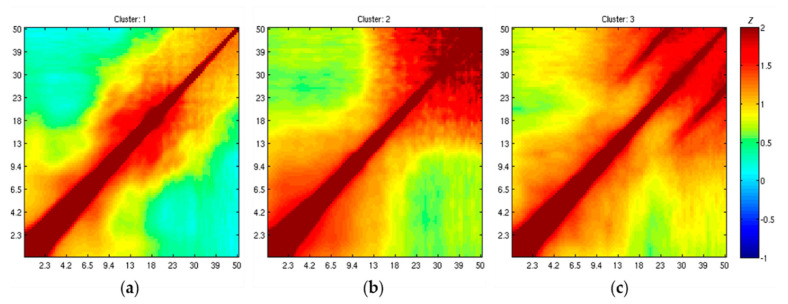
Matrices of standardized correlation for the different IC classes showing discernible patterns across the classes from highest-level analysis, where each of the standardized correlation is color-coded as represented in z scale: (**a**) The standardized matrix of brain class shows cross-frequency PPC between 6.5and 30 Hz and well-suppressed off-diagonal couplings; (**b**) the standardized matrix of eye class shows block-diagonalization on the lower end of the frequencies in 1-9.4 Hz and the higher end of the frequencies in 23–50 Hz; (**c**) the standardized matrix of eye class shows the characteristic parallel lines between 30 and 50 Hz.

The grand comodulograms of standardized correlation for brain activity, eye movement, and muscle activity IC classes are plotted below (Figure 2) to confirm the general impressions. The strong couplings and the diagonal lines represent self-correlation and should be ignored. The visual examination of these comodulograms indicates that (1) anti-correlation (i.e., *corr_ij_* < 0) was rare. (2) Differences across the classes are present: In the brain-activity IC class (left), the cross-frequency PPC was found between 6.5 and 30 Hz, and that in the off-diagonal area was weakly anti-correlated (see the blue color in the off-diagonal area); in the eye-movement IC class (Figure 2b), it showed characteristic block-diagonalization on the lower end of the frequencies in 1–9.4 Hz and the higher end of the frequencies in 23–50 Hz; lastly, in the muscle IC class (Figure 2c), characteristic *parallel lines* were observed between 30 and 50 Hz. In visual examination on PSD, such a high-frequency PPC is hard to find because they do not form a noticeable peak in the frequency domain. To sum, this highest-level result showed successful distinction at the level of visual inspection among the three classes of the EEG signals decomposed by ICA.

### 3.2. Representative Comodulogram of Brain Class

**Figure 3:**
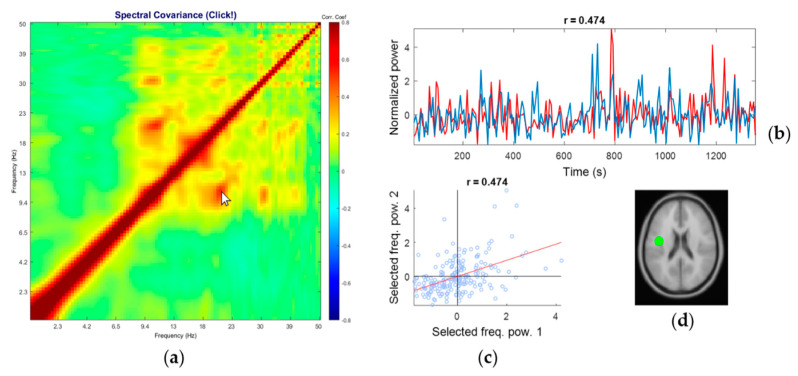
A representative comodulogram of Brain-Class (Subject 20, IC No. 26): (**a**) the comodulogram; (**b**) time series of the cross-frequency PPC of the combinations of the selected frequency bins specified by the cursor position on the comodulogram; the currently selected pair is 10.7 Hz and 21.3 Hz, and their correlation coefficient is 0.474; (**c**) the scatter plot of PSD from individual windows of the selected pair; (**d**) the corresponding equivalent current dipole model localized at (−43, −4, 27) in Talairach coordinates, which corresponds to Brodmann Area 6. Note the isolated islands of the red blobs with the regular intervals on the log scale, which indicates harmonics that are integer multiples of the fundamental frequency (i.e., alpha band).

Figure 3 shows an example of a representative comodulogram from the brain-class IC (the equivalent current dipole coordinate was (−43, −4, 27) in Talairach coordinates, localized within left Brodmann Area 6), its normalized time series of the selected pair (by cursor position on the main comodulogram plot) of frequencies $f_{i}$ and $f_{j}$, scatter plot of $f_{i}$ against $f_{j}$, and the corresponding equivalent current dipole location. The IC in Figure 3 is the same as in Figure 1. The comodulogram showed alpha harmonics up to 4th order, though the corresponding PSD showed only 2nd harmonic. Such a cross-frequency coupled alpha oscillation was reported in the visual experiment known as *resonance effects* in the visual cortex [57]. However, as far as we know, this is the first report of 4th order harmonic of the human alpha-band oscillation. The results demonstrated that the proposed method allows us to reveal a cross-frequency coupling structure not available for standard PSD by FFT.

### 3.3. Representative Comodulogram of Eye Class

**Figure 4:**
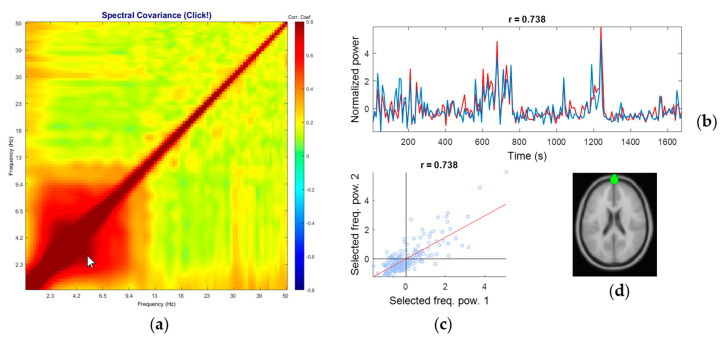
A representative comodulogram of Eye-Class (Subject 1, IC No. 1): (**a**) the comodulogram; (**b**) time series of the cross-frequency PPC of the combinations of the selected frequency bins specified by the cursor position on the comodulogram; the currently selected pair is 2.7 Hz and 5.3 Hz, and their correlation coefficient is 0.738; (**c**) the scatter plot of PSD from individual windows of the selected pair; (**d**) the corresponding equivalent current dipole model localized at (−1, 72, −5) in Talairach coordinates, which identified as eye artifact.

Figure 4 shows an example of a representative comodulogram from the eye-class IC (the equivalent current dipole coordinate was (−1, 72, −5) in Talairach coordinates, localized outside the brain). The eye-class characteristic is the broad, within-low-frequency coupling (delta and theta bands, below 9.4 Hz on the plot) that composes a square on the bottom left of the diagonal line. This is in line with the report that blinks and eye movements generate large electrical activities—relative to brain-source EEG—within delta and theta frequency ranges but not in the alpha range and above [58]. However, this feature is often hard to distinguish from the EEG signal’s 1/f distribution in PSD.

It is hard to distinguish frontal brain component from eye component just by referring to PSD because of these overlapping features. Comparing the comodulograms of the brain class and the eye class in Figure 2 and between Figure 3 and Figure 4, it is clear that PPC within the below-theta range is observed only in the eye class.

### 3.4. Representative Comodulogram of Muscle Class

**Figure 5:**
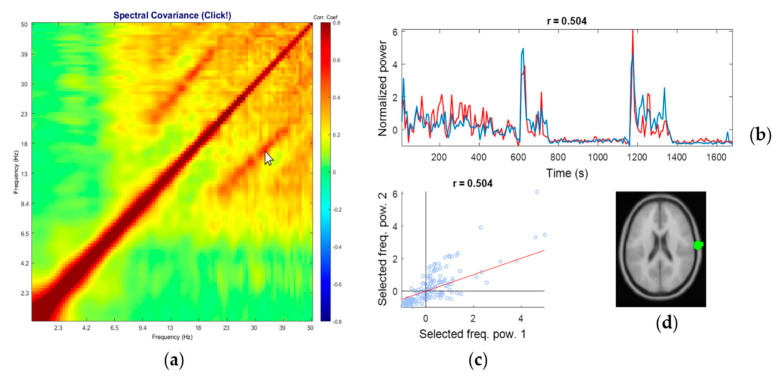
A representative comodulogram of Muscle-Class (Subject 3, IC No. 16): (**a**) the comodulogram; (**b**) time series of the cross-frequency PPC of the combinations of the selected frequency bins specified by the cursor position on the comodulogram; the currently selected pair is 16.0 Hz and 32.6 Hz, and their correlation coefficient is 0.504; (**c**) the scatter plot of PSD from individual windows of the selected pair; (**d**) the corresponding equivalent current dipole model localized at (72, −10, −6) in Talairach coordinates, which identified as muscle artifact. Note the broadband second harmonics as a *parallel line* between 10 and 20 Hz coupled with 20–40 Hz.

Figure 5 shows an example of a representative comodulogram from the muscle-class IC (the equivalent current dipole coordinate was (72, −10, −6) in Talairach coordinates, localized outside the brain). The comodulogram showed distinct parallel lines in the beta and gamma frequency ranges (20–40 Hz) as a second harmonic of 10–20 Hz, which is also visible in this class’s standardized comodulogram in Figure 2. The known fact is that PSD of muscle activity lies below 40 Hz though it extends to 300 Hz [59]. However, it is the first time to reveal that muscle activity has characteristic cross-frequency PPC continuously from 10–20 Hz (coupled with 20–40Hz). Figure 5b shows the time-series of PSD changes at 16.0 Hz and 32.6 Hz, which confirmed a strong correlation (r = 0.504). To sum, we found a characteristic comodulogram pattern unique to the muscle class.

### 3.5. IC Clustering, According to Comodulogram

**Figure 6:**
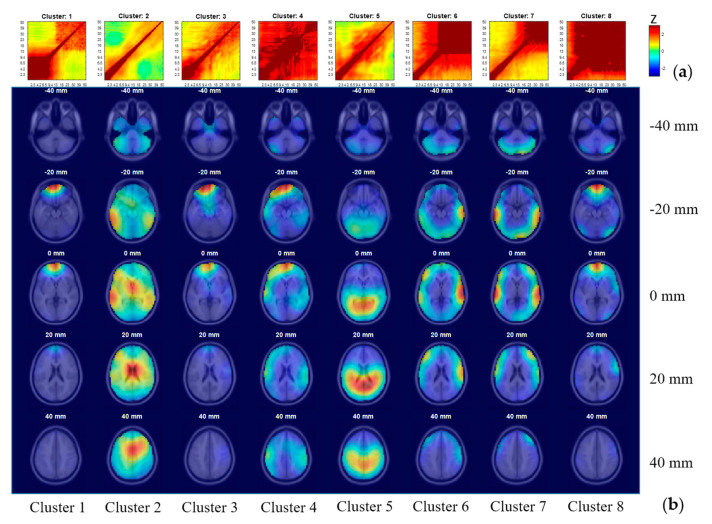
The results from comodulogram-based clustering. (**a**) The correlation matrices standardized in each cluster and represented in the right-panel z scale. (**b**) The dipole density maps projected on anatomical images in axial view at different MNI z coordinates ranging from 0 mm (mm) to 80 mm; warmer color represents a higher density of equivalent dipoles. The same k-means clustering was used with the number of clusters specified to be 8. Cluster 2 and Cluster 5 and potentially Cluster 3 as well were identified as the brain class. Cluster 1 was identified as the eye class. Cluster 6, Cluster 7, and Cluster 8 were identified as the muscle class. Cluster 4 was not classified to any of these categories, thus left as uninterpretable noise.

Figure 6 shows the standardized correlation matrices and the dipole density maps projected on anatomical images in axial view at different z coordinates in standard MNI space. Our visual inspection identified Cluster 2 as the brain class for the well-suppressed off-diagonal areas (near blue) and the frontocentral dipole density distribution. Cluster 5 was also identified as the brain class for the centroparietal dipole density distribution. However, Cluster 5’s comodulogram showed an increase of correlations between 10 and 30 Hz, which differs from that of Cluster 2. We concluded that the comodulogram-based clustering identified (at least) two separate clear brain classes. Cluster 3 and Cluster 4 showed some brain class evidence due to their weak frontal dipole density distribution, but they also suggest dipole distribution within the frontal the right temporal areas. Their comodulograms showed that Cluster 3 is similar to Cluster 2, but Cluster 4’s comodulogram is not. This qualitative evaluation concluded that Cluster 3 may consist of many brain sources, while Cluster 4 may consist of artifacts. Cluster 1 showed a block diagonal comodulogram centered on the low frequency (<9.4 Hz). From what we learned above, we identified this cluster as the eye class. The dipole density distribution of the cluster supported this interpretation. Cluster 6, Cluster 7, and Cluster 8 showed block diagonal comodulograms centered on the higher frequency (above 13, 18, and 4 Hz, respectively). From our observations above, we identified these clusters as the muscle class. Dipole density distribution also showed peripheral distributions.

Further, there is a tendency that lower *z* coordinates are associated with temporo-occipital distribution, while higher *z* coordinates are related to frontal distributions. This makes sense in terms of the scalp electrode locations in which the edge channels tend to pick up muscle potentials. To sum, the comodulogram-based clustering produced reasonable classifications of the data supported by the estimated source distributions and their functions.

In the test of suitability of using comodulogram-based clustering to classify ICs, we evaluated the consistency between our interpretation on each cluster as explained above and the majority class of ICs in that cluster as labeled by ICLabel. Table 1 shows the results. In Clusters 1, 5, and 6, more than 80% of ICs were classified into a single class, showing the high consistency level between comodulogram method and ICLabel. All ICs from these clusters are combined to represent 32.7% (258 ICs) of total ICs. The classes with maximal proportion are also consistent with our interpretation. Meanwhile, Clusters 2, 4, 7, and 8 show reasonable consistency levels between 0.5 and 0.8, showing moderate success. These clusters constitute 60.3% (476 ICs) of total ICs. The majority of ICs in Cluster 2 were classified into brain class. As we expected, clusters 7 and 8 had most ICs classified as muscle. However, we considered Cluster 4 as uninterpretable noise. This cluster had many eye ICs, demonstrating the limitation of comodulogram-based clustering. Nevertheless, this cluster with 30 ICs constituted to only 3.8% of the total ICs. The remaining ICs in the cluster were approximately evenly classified into brain, muscle, and eye class, making this cluster inconclusive. The consistent level was limited to 26.8%, but this cluster constitutes only 7.1% (56 ICs) of the total ICs. To sum up, even simple k-means can successfully categorize ICs with similar properties into reasonably separate subgroups. Around one-third of total ICs can be classified correctly with impressive consistency level (all > 82%) with ICLabel (an SCCN/EEGLAB plugin). The majority of remaining clusters are classified with a reasonable consistency level (all > 51.5%) than the chance level of 42.5%. We concluded that the results demonstrate the usefulness of comodulogram PPC for IC classification.

## 4. Discussion

In the present study, we demonstrated the usefulness of cross-frequency PPC analysis on ICA-decomposed EEG data. We showed that the brain, eye, and muscle classes have their features in comodulograms. The classification from comodulogram-based clustering was supported by physiological validity provided by their estimated source distributions and the reasonable consistency with the results from a standardized IC classification tool (ICLabel).

From the analysis of representative ICs, we found that the proposed method allows us to reveal a cross-frequency coupling structure unavailable for PSD by FFT. Besides, there exist many unique characteristics among brain, eye-movement, and muscle activity classes, which have been overlooked so far. PPC is a unique metric and is an added value for EEG IC classifications. We demonstrated that comodulogram-based clustering using a simple k-means algorithm yields satisfactory results. Using more sophisticated supervised/unsupervised learning with fine-tuned PPC calculation is an easy target toward better IC classification performance.

**Figure 7:**
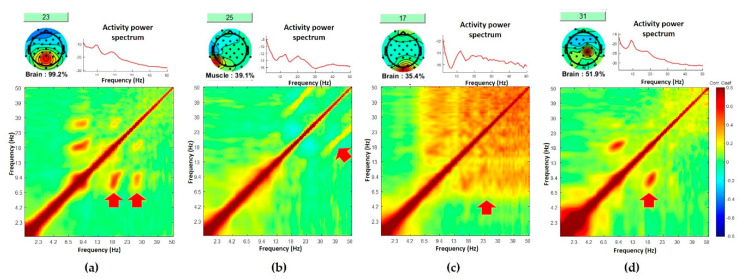
For each column, a class label suggested by ICLabel (top left, below scalp topography), power spectral density (top right), and comodulogram, i.e., cross-frequency PPC plot (bottom): (**a**) results from Subject 1 show the concordance between ICLabel and PPC results; (**b**) results from Subject 2 show the occurrence of a parallel line in beta–gamma coupling in possibly an EMG IC which is consistent with the label from ICLabel despite low label probability; (**c**) data from Subject 3 led to the discordance between “Brain” label suggested by ICLabel and comodulogram pattern unique to the muscle class indicated by the current study; (**d**) the presence of the alpha harmonics in comodulogram from Subject 4 can boost the label probability of ICLabel-suggested class label as “Brain” despite the current low likelihood.

In this study, we evaluated our classification’s consistency via three methods: first, by comparing our results with classification labels generated by ICLabel; second, by visual examination of PPC clustering results; and third, by examining the PPC clustering results using a standardized comodulogram and the probabilistic dipole density. Our comodulogram-based clustering was judged against the operational *ground truths* for the current purpose generated by ICLabel. While label probabilities calculated by ICLabel are typically very high (mostly > 80%), sometimes the highest label probability can be no more than 50%. When we examined individual ICs with low label probability, we often found that comodulogram can inform us more about the ICs with additional insight. Examples from representative ICs selected from four different subjects are illustrated in Figure 7. These plots show PSD, the class label suggested by ICLabel with label probability, and the corresponding comodulogram. In the case of successful prediction in Figure 7a, the Brain class shows convincing evidence of the first and second harmonics of alpha rhythm. In contrast, the Muscle class in Figure 7b has low label probability of 0.391, but the corresponding comodulogram shows clear parallel lines in the beta–gamma coupling, which is the common pattern we found for the case of muscle class. The ICLabel-generated label of “Brain” in Figure 7c seems challenged by high-frequency broadband coupling, which is a unique characteristic of muscle ICs. Finally, comodulogram in Figure 7d clearly shows the presence of the first alpha harmonic, despite a relatively low label probability of 0.519. These examples demonstrate that PPC comodulogram can deliver complementary insights about ICs. It might help improve the classification of ICs, especially when used together with traditional methods, thus opening new possibilities for better data-driven EEG data classification. A future systematic study may test this possibility.

Note that any correlation measure is susceptible to the presence of extreme values; data with non-normal distributions require cautious use of correlational measures [60]. In the current application, muscle-class ICs showed higher powers and more sporadic activations over time compared with constant and stationary brain-class ICs. We indeed observed that the intermittent, high-power muscle activity calculated comodulogram was often unsuccessful. After investigation, we learned that extensive removal of outliers is essential for successful computation. Using robust statistics, we empirically determined trimming the top and bottom 10% from the data distribution for data cleaning resulted in satisfactory outcomes. However, we are dissatisfied this approach provides neither theoretical nor qualitative solutions to the problem. Future studies must address outliers in calculating comodulograms.

From our earlier review, we observed cross-frequency PPC had been used more in the animal electrophysiological studies using LFP. In contrast, we show that the comodulogram measure alone can classify effective EEG sources into representative physiological categories. This evidence strongly supports generalization of the PPC within EEG analyses across disparate fields. We await future studies that will clarify the generative mechanism of the cross-frequency PPC for the physiological classes (brain, eye, and muscle), sub-categories of the brain (i.e., perceptual, cognitive, behavioral functions), as well as anatomical regions. Such studies would make PPC electro-physiologically informed physiologically established measure.

## 5. Conclusions

We demonstrated that brain, eye, and muscle classes of ICA-decomposed scalp-recorded EEG signals have characteristic features in comodulograms of cross-frequency PPC analysis. The classification based on the comodulogram was supported by physiological validity provided by the estimated source distribution and the reasonable consistency with the results from a standardized IC classification tool. The cross-frequency PPC measure is a new, data-driven EEG-IC classifier capable of exploiting underrepresented univariate single-channel/component time-frequency structure information. The data processing methods herein and the interactive visualization searching environment are freely available as open-source Matlab library plugins in EEGLAB [42] to facilitate researchers’ replication of our analysis.

## Figures and Tables

**Table 1:** Percentage of independent components (ICs) in each class (as labeled by ICLabel to the total number of ICs in each cluster; note that the clusters were reordered to enhance readability.

Clusters	Our Interpretation	Percentage of ICs in Each Class as Labeled by ICLabel							Total Number of ICs
		Brain	Muscle	Eye	Heart	Line Noise	Channel Noise	Other	
2	Brain	**52.5**	23.0	1.8	0.0	13.5	0.0	9.2	282
3	Brain	23.2	**26.8**	16.1	0.0	25.0	0.0	8.9	56
5	Brain	**82.0**	8.7	0.0	0.0	8.7	0.0	0.7	150
6	Muscle	10.4	**83.1**	0.0	0.0	2.6	0.0	3.9	77
7	Muscle	27.6	**51.5**	2.2	0.0	5.2	0.0	13.4	134
8	Muscle	3.3	**53.3**	26.7	0.0	10.0	0.0	6.7	30
1	Eye	3.2	0.0	**83.9**	0.0	6.5	0.0	6.5	31
4	Noise	16.7	20.0	**56.7**	0.0	3.3	3.3	0.0	30
Total		42.5	31.4	8.6	0.0	10.1	0.1	7.2	790

## References

[1] Sterman M.B., Kaiser D. (2000). Comodulation: A new qEEG analysis metric for assessment of structural and functional disorders of the central nervous system. J. Neurother..

[2] Gaidica M., Hurst A., Cyr C., Leventhal D.K. (2020). Interactions Between Motor Thalamic Field Potentials and Single-Unit Spiking Are Correlated with Behavior in Rats. Front. Neural Circuits.

[3] Senzai Y., Fernandez-Ruiz A., Buzsáki G. (2019). Layer-Specific Physiological Features and Interlaminar Interactions in the Primary Visual Cortex of the Mouse. Neuron.

[4] Khodagholy D., Gelinas J.N., Buzsáki G. (2017). Learning-enhanced coupling between ripple oscillations in association cortices and hippocampus. Science.

[5] Leventhal D.K., Gage G.J., Schmidt R., Pettibone J.R., Case A.C., Berke J.D. (2012). Basal Ganglia Beta Oscillations Accompany Cue Utilization. Neuron.

[6] Conde-Moro A.R., Rocha-Almeida F., Sánchez-Campusano R., Delgado-García J.M., Gruart A. (2019). The activity of the prelimbic cortex in rats is enhanced during the cooperative acquisition of an instrumental learning task. Prog. Neurobiol..

[7] Watson B.O., Levenstein D., Greene J.P., Gelinas J.N., Buzsáki G. (2016). Network Homeostasis and State Dynamics of Neocortical Sleep. Neuron.

[8] Zhou Y., Sheremet A., Qin Y., Kennedy J.P., DiCola N.M., Maurer A.P. (2019). High-order theta harmonics account for the detection of slow gamma. eNeuro.

[9] Buzsáki G., Buhl D., Harris K., Csicsvari J., Czéh B., Morozov A. (2003). Hippocampal network patterns of activity in the mouse. Neuroscience.

[10] Buzsáki G., Wang X.J. (2012). Mechanisms of gamma oscillations. Annu. Rev. Neurosci..

[11] Quilichini P., Sirota A., Buzsáki G. (2010). Intrinsic circuit organization and theta–gamma oscillation dynamics in the entorhinal cortex of the rat. J. Neurosci..

[12] Sullivan D., Csicsvari J., Mizuseki K., Montgomery S., Diba K., Buzsáki G. (2011). Relationships between hippocampal sharp waves, ripples, and fast gamma oscillation: Influence of dentate and entorhinal cortical activity. J. Neurosci..

[13] Stujenske J., Likhtik E., Topiwala M., Gordon J. (2014). Fear and safety engage competing patterns of theta-gamma coupling in the basolateral amygdala. Neuron.

[14] Ferraris M., Ghestem A., Vicente A.F., Nallet-Khosrofian L., Bernard C., Quilichini P.P. (2018). The Nucleus Reuniens Controls Long-Range Hippocampo–Prefrontal Gamma Synchronization during Slow Oscillations. J. Neurosci..

[15] Shirvalkar P.R., Rapp P.R., Shapiro M.L. (2010). Bidirectional changes to hippocampal theta–gamma comodulation predict memory for recent spatial episodes. Proc. Natl. Acad. Sci. USA.

[16] Carlqvist H., Nikulin V.V., Strömberg J.O., Brismar T. (2005). Amplitude and phase relationship between alpha and beta oscillations in the human electroencephalogram. Med. Biol. Eng. Comput..

[17] Llinás R.R., Ribary U., Jeanmonod D., Kronberg E., Mitra P.P. (1999). Thalamocortical dysrhythmia: A neurological and neuropsychiatric syndrome characterized by magnetoencephalography. Proc. Natl. Acad. Sci. USA.

[18] Mitra P., Bokil H. (2007). Observed Brain Dynamics.

[19] Llinás R.R., Ribary U., Jeanmonod D. (2004). Method and System for Diagnosing and Treating Thalamocortical Dysrhythmia. U.S. Patent.

[20] Masimore B., Kakalios J., Redish A.D. (2004). Measuring fundamental frequencies in local field potentials. J. Neurosci. Methods.

[21] Schultheiss N.W., Schlecht M., Jayachandran M., Brooks D.R., McGlothan J.L., Guilarte T.R., Allen T.A. (2020). Awake delta and theta-rhythmic hippocampal network modes during intermittent locomotor behaviors in the rat. Behav. Neurosci..

[22] Zhou Y., Sheremet A., Qin Y., Kennedy J.P., DiCola N.M., Burke S.N., Maurer A.P. (2019). Methodological Considerations on the Use of Different Spectral Decomposition Algorithms to Study Hippocampal Rhythms. eNeuro.

[23] Sheremet A., Kennedy J.P., Qin Y., Zhou Y., Lovett S.D., Burke S.N., Maurer A.P. (2019). Theta-gamma cascades and running speed. J. Neurophysiol..

[24] Popov T., Jensen O., Schoffelen J.-M. (2018). Dorsal and ventral cortices are coupled by cross-frequency interactions during working memory. NeuroImage.

[25] Wang L., Hagoort P., Jensen O. (2018). Language prediction is reflected by coupling between frontal gamma and posterior alpha oscillations. J. Cogn. Neurosci..

[26] Bell A.J., Sejnowski T.J. (1995). An information-maximization approach to blind separation and blind deconvolution. Neural Comput..

[27] Makeig S., Bell A.J., Jung T.P., Sejnowski T.J. (1996). Independent component analysis of electroencephalographic data. Advances in Neural Information Processing Systems 8.

[28] Makeig S., Jung T.P., Bell A.J., Ghahremani D., Sejnowski T.J. (1997). Blind separation of auditory event-related brain responses into independent components. Proc. Natl. Acad. Sci. USA.

[29] Makeig S., Westerfield M., Jung T.P., Enghoff S., Townsend J., Courchesne E., Sejnowski T.J. (2002). Dynamic brain sources of visual evoked responses. Science.

[30] Onton J., Makeig S. (2006). Information-based modeling of event-related brain dynamics: Why use ICA to decompose EEG/MEG data?. Prog. Brain Res..

[31] Winkler I., Haufe S., Tangermann M. (2011). Automatic classification of artifactual ICA-components for artifact removal in EEG signals. Behav. Brain Funct..

[32] Winkler I., Brandl S., Horn F., Waldburger E., Allefeld C., Tangermann M. (2014). Robust artifactual independent component classification for bci practitioners. J. Neural Eng..

[33] Mognon A., Jovicich J., Bruzzone L., Buiatti M. (2011). Adjust: An automatic EEG artifact detector based on the joint use of spatial and temporal features. Psychophysiology.

[34] Nolan H., Whelan R., Reilly R. (2010). Faster: Fully automated statistical thresholding for EEG artifact rejection. J. Neurosci. Methods.

[35] Bigdely-Shamlo N., Kreutz-Delgado K., Kothe C., Makeig S. Eyecatch: Data-mining over half a million EEG independent components to construct a fully-automated eye-component detector. Proceedings of the 2013 35th Annual International Conference of the IEEE Engineering in Medicine and Biology Society EMBC.

[36] Frølich L., Andersen T.S., Mørup M. (2015). Classification of independent components of EEG into multiple artifact classes. Psychophysiology.

[37] Chaumon M., Bishop D.V., Busch N.A. (2015). A practical guide to the selection of independent components of the electroencephalogram for artifact correction. J. Neurosci. Methods.

[38] Pion-Tonachini L., Kreutz-Delgado K., Makeig S. (2019). ICLabel: An automated electroencephalographic independent component classifier, dataset, and website. NeuroImage.

[39] Viola F.C., Thorne J., Edmonds B., Schneider T., Eichele T., Debener S. (2009). Semi-automatic identification of independent components representing EEG artifact. Clin. Neurophysiol..

[40] Gabsteiger F., Leutheuser H., Reis P., Lochmann M., Eskofier B.M. (2014). SVM for Semi-automatic Selection of ICA Components of Electromyogenic Artifacts in EEG Data. Proceedings of the 15th International Conference on Biomedical Engineering, Singapore, 4–7 December 2013.

[41] Makeig S. (1993). Auditory event-related dynamics of the EEG spectrum and effects of exposure to tones. Electroencephalogr. Clin. Neurophysiol..

[42] Delorme A., Makeig S. (2004). EEGLAB: An open source toolbox for analysis of single-trial EEG dynamics including independent component analysis. J. Neurosci. Methods.

[43] Miyakoshi M., Kanayama N., Iidaka T., Ohira H. (2010). EEG evidence of face-specific visual self-representation. NeuroImage.

[44] Nunez P.L., Srinivasan R. (2007). Electric Fields of the Brain: The Neurophysics of EEG.

[45] Widmann A., Schröger E., Maess B. (2015). Digital filter design for electrophysiological data—A practical approach. J. Neurosci. Methods.

[46] Palmer J.A., Makeig S., Kreutz-Delgado K., Rao B.D. Newton method for the ICA mixture model. Proceedings of the 2008 IEEE International Conference on Acoustics, Speech and Signal Processing.

[47] Delorme A., Palmer J., Onton J., Oostenveld R., Makeig S. (2012). Independent EEG Sources Are Dipolar. PLoS ONE.

[48] Oostenveld R., Fries P., Maris E., Schoffelen J.M. (2011). Fieldtrip: Open source software for advanced analysis of MEG, EEG, and invasive electrophysiological data. Comput. Intell. Neurosci..

[49] Muller L., Piantoni G., Koller D., Cash S.S., Halgren E., Sejnowski T.J. (2016). Rotating waves during human sleep spindles organize global patterns of activity that repeat precisely through the night. eLife.

[50] Rissling A.J., Miyakoshi M., Sugar C.A., Braff D.L., Makeig S., Light G.A. (2014). Cortical substrates and functional correlates of auditory deviance processing deficits in schizophrenia. NeuroImage Clin..

[51] Makeig S., Westerfield M., Townsend J., Jung T.-P., Courchesne E., Sejnowski T.J. (1999). Functionally independent components of early event-related potentials in a visual spatial attention task. Philos. Trans. R. Soc. B Biol. Sci..

[52] Makeig S., Debener S., Onton J., Delorme A. (2004). Mining event-related brain dynamics. Trends Cogn. Sci..

[53] Groppe D.M., Makeig S., Kutas M. (2009). Identifying reliable independent components via split-half comparisons. Neuroimage.

[54] Jamšek J., Paluš M., Stefanovska A. (2010). Detecting couplings between interacting oscillators with time-varying basic frequencies: Instantaneous wavelet bispectrum and information theoretic approach. Phys. Rev. E.

[55] Lancaster G., Iatsenko D., Pidde A., Ticcinelli V., Stefanovska A. (2018). Surrogate data for hypothesis testing of physical systems. Phys. Rep..

[56] Stankovski T., Ticcinelli V., McClintock P.V.E., Stefanovska A. (2017). Neural Cross-Frequency Coupling Functions, Frontiers in Systems. Neuroscience.

[57] Herrmann C.S. (2001). Human EEG responses to 1–100 Hz flicker: Resonance phenomena in visual cortex and their potential correlation to cognitive phenomena. Exp. Brain Res..

[58] Hagemann D., Naumann E. (2001). The effects of ocular artifacts on (lateralized) broadband power in the EEG. Clin. Neurophysiol..

[59] Criswell E. (2010). Cram’s Introduction to Surface Electromyography.

[60] Anscombe F.J. (1973). Graphs in statistical analysis. Am. Stat..

